# Japanese Nationwide Study on the Association Between Short-term Exposure to Particulate Matter and Mortality

**DOI:** 10.2188/jea.JE20180122

**Published:** 2019-12-05

**Authors:** Takehiro Michikawa, Kayo Ueda, Akinori Takami, Seiji Sugata, Ayako Yoshino, Hiroshi Nitta, Shin Yamazaki

**Affiliations:** 1 Centre for Health and Environmental Risk Research, National Institute for Environmental Studies, Ibaraki, Japan; 2 Department of Environmental and Occupational Health, School of Medicine, Toho University, Tokyo, Japan; 3 Environmental Health Sciences, Kyoto University Graduate School of Global Environmental Studies, Kyoto, Japan; 4 Department of Environmental Engineering, Graduate School of Engineering, Kyoto University, Kyoto, Japan; 5 Centre for Regional Environmental Research, National Institute for Environmental Studies, Ibaraki, Japan

**Keywords:** fine particulate matter, coarse particle, mortality, case-crossover, Japanese

## Abstract

**Background:**

From around 2012, the use of automated equipment for fine particulate matter (PM_2.5_) measurement with equivalence to a reference method has become popular nationwide in Japan. This enabled us to perform a national health effect assessment employing PM_2.5_ concentrations based on the standardized measurement method. We evaluated the association between non-accidental mortality and short-term exposure to PM_2.5_ and coarse particulate matter (PM), with the latter estimated as the difference between suspended particulate matter and PM_2.5_, for the fiscal years 2012–2014.

**Methods:**

This was a time-stratified case-crossover study in 100 highly-populated Japanese cities. Mortality data was obtained from the Ministry of Health, Labour and Welfare. City-specific estimates of PM-mortality association were calculated by applying a conditional logistic regression analysis, and combined with a random-effects meta-analysis.

**Results:**

The respective averages of daily mean concentration were 14.6 µg/m^3^ for PM_2.5_ and 6.4 µg/m^3^ for coarse PM. A 10 µg/m^3^ increase in PM_2.5_ concentrations for the average of the day of death and the previous day was associated with an increase of 1.3% (95% confidence interval (CI), 0.9–1.6%) in total non-accidental mortality. For cause-specific mortality, PM_2.5_ was positively associated with cardiovascular and respiratory mortality. After adjustment for PM_2.5_, we observed a 1.4% (95% CI, 0.2–2.6%) increase in total mortality with a 10 µg/m^3^ increase in coarse PM.

**Conclusion:**

The study revealed that short-term exposure to PM_2.5_ had adverse effects on total non-accidental, cardiovascular, and respiratory mortality in Japan. Coarse PM exposure also increased the risk of total mortality.

## INTRODUCTION

The health effects of particulate matter, particularly fine particulate matter (PM_2.5_ that passes through a size-selective inlet with a 50% cut-off level of 2.5 µm in aerodynamic diameter), are an international concern,^1^ and epidemiologic evidence increases year by year. For instance, with respect to mortality as a classical outcome, a PM_2.5_-daily mortality association has been established, and the effect size, on a national or multi-country scale, has been considered from the viewpoint of public health. One nationwide study in 112 American cities revealed that an increase of 10 µg/m^3^ in PM_2.5_ exposure was associated with a 0.98% increase in all-cause mortality,^2^ and another nationwide study, in 272 Chinese cities, estimated a similar increase of 0.22% in mortality.^3^ Further, a multi-country study in 10 European cities estimated a 0.55% increase in mortality.^4^ Nonetheless, evidence of the effects of PM_2.5_ exposure on health in Japan is extremely limited because the routine measurement of PM_2.5_ in Japanese air pollution monitoring stations only began after the Air Quality Standard for PM_2.5_ was established in 2009.^5^ Previously, we reported that short-term exposure to PM_2.5_ was positively associated with all-cause mortality in those aged 65 years or older in 20 regions between 2002 and 2004^6^; however, PM_2.5_ concentration measurement equivalence was not ensured among the 20 automated measuring devices in the study.

To perform PM_2.5_ concentration comparison among cities, systematic measurement error based on the measuring equipment should be avoided. Therefore, the Japanese Air Quality Standards have defined the PM_2.5_ measurement method as follows: “mass measurement with filter sample collection, which is designated as a reference method, or alternative automated methods, designated as equivalent methods, which are proved to have measurement performance comparable to the corresponding reference method”.^5^ From around 2012, use of automated equipment enabling PM_2.5_ measurement with equivalence to a reference method has become popular nationwide. This allowed us to perform a national health effect assessment that employed PM_2.5_ concentrations based on the standardized measurement method.

We conducted a multi-city case-crossover study of the association between short-term exposure to PM_2.5_ and mortality in Japan. Additionally, with regard to exposure to coarse particulate matter (coarse PM), for which there is still limited health effects evidence globally, we explored the association with mortality using an exposure surrogate for coarse PM, estimated as the difference between the concentrations of suspended particulate matter (SPM that passes through a size-selective inlet with a 100% cut-off level of 10 µm in aerodynamic diameter) and PM_2.5_.

## METHODS

### Study area

In the 2015 Japanese Population Census, there were 110 cities with a total population exceeding 200,000. Of these 110 cities, 99 had one or more ambient air pollution monitoring stations with automated PM_2.5_ measuring equipment with measurement equivalence to the reference method. Additionally, we included in the study area Tsukuba city (population: roughly 227,000 people in the 2015 Population Census), which has no monitoring station but is the home of the Japan National Institute for Environmental Studies, by combining Tsukuba City and adjacent Tsuchiura City (which has a monitoring station) into one city. Our 100 target cities were located throughout the length of Japan, from Hokkaido Prefecture (Sapporo, Asahikawa, and Hakodate) to Okinawa Prefecture (Naha). The population and size of the 100 target cities are presented in eTable 1. The study protocol was reviewed and approved by the Ethics Committee of the National Institute for Environmental Studies (2017-004), and the Faculty of Medicine, Toho University (A18051).

### Environmental data

The hourly measurement of PM_2.5_ concentrations using the reference method-equivalent automated PM_2.5_ measuring equipment, measured at one ambient air pollution monitoring station within each city from April 2012 to March 2015, were obtained from the Japan National Institute for Environmental Studies’ atmospheric environment database. The PM_2.5_ measurement periods varied by city (eTable 1). Among 100 cities, 66 cities had only one ambient air monitoring station measuring PM_2.5_. According to the pollutant monitoring manual of the Ministry of the Environment, Japan,^7^ such stations are located at the points that seem to be representative of pollutant concentrations in the community. For the remaining cities, which all had two or more ambient air pollution monitoring stations, we selected one that satisfied the following criteria: (1) the station measured other co-pollutants, including SPM, photochemical oxidants (Ox), nitrogen dioxide (NO_2_), and sulphur dioxide (SO_2_), in addition to PM_2.5_; and/or (2) the station was located nearer to the center of the city than other stations. However, in such cases, we confirmed a strong correlation between the PM_2.5_ concentrations measured at the selected station and those of the other station(s) in the same city (mean correlation coefficient = 0.95). The locations of the monitoring stations in the 100 target Japanese cities are presented in eFigure 1. The daily mean concentrations of PM_2.5_ were calculated based on hourly measurements from 0 to 23 h, and no daily mean concentrations were calculated for days when more than four hourly measurements were missing. The median % missing days in the PM_2.5_ data was 1.2% (interquartile range [IQR], 0.6–2.2%), and these days were excluded in the analysis.

The hourly measurements of other co-pollutants were also obtained, and the daily mean concentrations of SPM, NO_2_ and SO_2_, as well as the maximum 8-h concentrations of Ox, were calculated. In this study, the difference between the respective concentrations of SPM and PM_2.5_ was used as an exposure surrogate for coarse PM. Internationally, coarse PM is defined as particulate matter measuring between 2.5 µm and 10 µm (PM_10-2.5_) in diameter.^8^ However, the monitoring stations in Japan do not routinely measure PM_10_ (particles that pass through a size-selective inlet with a 50% cut-off level of 10 µm in aerodynamic diameter). For the coarse PM-mortality association, we included 77 cities where concentrations of coarse PM had been reasonably distributed.

Meteorological data (daily mean ambient temperature and relative humidity), measured at the nearest meteorological observatory to each city, were collected from the Japan Meteorological Agency. Finally, information on the weekly influenza counts was collected from the Japan National Institute of Infectious Diseases, and an influenza epidemic was considered to persist for the number of weeks above the 90 percentile of the distribution during the study period.^9^

### Mortality data

For all the study areas, we applied to the Ministry of Health, Labour and Welfare, Japan, for use of the data from its Vital Statistic Survey, and obtained daily mortality records, including the age and sex of the deceased, and the date and location of death, as well as the primary cause of death according to the 10^th^ edition of the International Classification of Diseases (ICD-10). In this study, our primary outcome was total non-accidental death (ICD-10: A00 through R99), and specific causes were defined as: cardiovascular disease (I00 through I99); coronary heart disease (I20 through I25); stroke (I60 through I69); and respiratory disease (J00 through J99).

### Statistical analysis

We used a time-stratified case-crossover design and compared an individual’s exposure on the “case” day with his or her exposure on the “control” days, when he or she did not become a case.^10^ We defined the “case” day as the day of death, and selected three or four “control” days from the same days of the week, in the same month and year, as the “case” day. For instance, if an individual died on Mar 15, 2013, four control days were assigned: March 1, 8, 22, and 29 of that year. We excluded cases on national holidays (not including weekends), to avoid bias in selection control due to the difference between pollutant concentrations on holidays and on non-holidays.

To investigate the association between exposure to PM_2.5_ and mortality, we first applied city-specific conditional logistic regression models. Based on past studies,^2^^,^^3^^,^^11^ the choice of the lag0-1 (2-day moving average of the case-day and previous-day) concentrations of PM_2.5_ was made before commencement of the study. The PM_2.5_ concentrations at lag2 and lag3 were used to investigate the lag pattern of the PM_2.5_-mortality association. We constructed a distributed lag model, simultaneously including PM_2.5_ concentrations at lag0-1, lag2, and lag3, and adjusted for ambient temperature at lag0-1 using a five-knot natural cubic spline, relative humidity at lag0-1 using a three-knot cubic spline, and influenza epidemics. Odds ratios (ORs) with 95% confidence intervals (CIs) of mortality based on an increase of 10 µg/m^3^ in PM_2.5_ concentration at lag0-1 were estimated because a linear model was not inferior to a non-linear model with respect to estimation of the PM_2.5_-mortality association. Subsequently, the city-specific estimates were used to obtain pooled estimates on the PM_2.5_-mortality association using random-effects meta-analysis. The ORs were converted into excess risks approximated by [(OR − 1) × 100]. The proportion of total variation in the city-specific estimates, attributable to heterogeneity, was assessed by computing the I^2^ statistic. The same two-stage approach was followed in estimating the association between coarse PM and mortality. We mutually adjusted for PM_2.5_ and coarse PM after estimating each PM-mortality association in the single-pollutant model.

For sensitivity analysis, we constructed two-pollutant models; and another model additionally adjusted for temperature at lag2-21 to investigate the long delay of the cold effects.^12^ With respect to PM_2.5_ exposure, to estimate the health effects at PM_2.5_ concentrations not exceeding the daily World Health Organization (WHO) guideline values,^13^ we analyzed after restricting to days with PM_2.5_ concentrations at lag0-1 ≤25 µg/m^3^. We also performed stratified analyses by age (<75 or ≥75 years) and sex, and examined seasonal variation in the PM-mortality association. Since the health effects of PM_2.5_ seem likely to differ with the composition and sources of PM_2.5_,^14^ we also explored region-specific associations: East Japan, where domestic pollution is the major contributor to PM_2.5_ concentrations; Central Japan; and West Japan, where transboundary pollution makes a large contribution to PM_2.5_ (eTable 1).^15^ All analyses were performed using STATA version 14 (Stata Corporation, College Station, TX, USA).

## RESULTS

In this nationwide analysis, the average daily mean PM_2.5_ concentration was 14.6 (standard deviation [SD], 8.3) µg/m^3^ (Table 1), and the 98th percentile of daily mean concentrations was 37.8 µg/m^3^. In terms of region-specific statistics, the respective averages of PM_2.5_ concentration were 13.6 (SD, 8.0) µg/m^3^ in East Japan, 14.6 (SD, 8.3) µg/m^3^ in Central Japan, and 17.9 (SD, 9.5) µg/m^3^ in West Japan. The average daily mean concentration of coarse PM was 6.4 (SD, 5.1) µg/m^3^. The city-specific results for environmental factors are presented in eTable 1. The Pearson’s correlation coefficients with PM_2.5_ were 0.33 for coarse PM, 0.37 for Ox, 0.47 for NO_2_, and 0.42 for SO_2_ (eTable 2). Among 1,347,152 non-accidental deaths, cardiovascular mortality accounted for 28.1% and respiratory mortality for 16.7% (Table 2).

**Table 1.** Overall statistics of environmental factors in 100 Japanese cities, April 2012 to March 2015

Environmental factors, daily mean	Number of cities	Mean (SD)	Percentile		

			25th	50th	75th
PM_2.5_, µg/m^3^	100	14.6 (8.3)	8.6	13.1	18.8
Coarse PM, µg/m^3 a^	77	6.4 (5.1)	2.9	5.6	8.9
Ox, ppb^b^	95	42.3 (15.2)	31.8	40.6	51.7
NO_2_, ppb	99	12.4 (6.1)	7.9	11.2	15.7
SO_2_, ppb	91	2.1 (1.2)	1.3	1.9	2.7
Ambient temperature, °C	100	15.7 (8.5)	7.7	16.0	23.0
Relative humidity, %	100	67 (13)	58	68	76

NO_2_, nitrogen dioxide; Ox, photochemical oxidants; PM, particulate matter; SD, standard deviation; SO_2_, sulphur dioxide.

^a^Concentrations of coarse PM were calculated by subtracting PM_2.5_ concentrations from those of suspended particulate matter.

^b^Daily maximum 8-h mean concentrations.

**Table 2.** Overall characteristics of non-accidental deaths (1,347,152 deaths) in 100 Japanese cities, April 2012 to March 2015

Outcome		Percent
Total non-accidental (ICD-10: A00–R99)		100
Age, years	<75	27.9
	≥75	72.1
Sex	Men	51.9
	Women	48.1
Cardiovascular disease (ICD-10: I00–99)		28.1
Coronary heart disease (ICD-10: I20–25)		6.4
Stroke (ICD-10: I60–69)		9.2
Respiratory disease (ICD-10: J00–99)		16.7

ICD, international classification of diseases.

When we explored the city-specific association between exposure to PM_2.5_ and total non-accidental mortality, the point estimates of percentage increase for 10 µg/m^3^ increase in PM_2.5_ at lag0-1 showed a direction of increased mortality risk in 72 cities (eFigure 2). We did not observe significant heterogeneity (I^2^ = 9.8%). Pooled estimates for the association between PM_2.5_ exposure and mortality are presented in Table 3. Exposure to PM_2.5_ at lag0-1 was associated with an increase in total mortality of 1.3% per 10 µg/m^3^ (95% CI, 0.9–1.6). The respective percentage increases for the association of exposure to PM_2.5_ and cause-specific mortality were 1.6% (95% CI, 0.8–2.4%) for cardiovascular disease, 2.7% (95% CI, 1.0–4.4%) for coronary heart disease, 1.3% (95% CI, −0.2 to 2.8%) for stroke, and 1.5% (95% CI, 0.7–2.3%) for respiratory disease. PM_2.5_ at lag2 and lag3 was not associated with either total or cause-specific mortality.

**Table 3.** Pooled estimates for the association between PM_2.5_ exposure and mortality in 100 Japanese cities, April 2012 to March 2015

Outcome	Lag0-1		Lag2		Lag3	
		
	Percentage increase for 10 µg/m^3^ increase in PM_2.5_^a^	(95% CI)	Percentage increase for 10 µg/m^3^ increase in PM_2.5_^a^	(95% CI)	Percentage increase for 10 µg/m^3^ increase in PM_2.5_^a^	(95% CI)
Total non-accidental (ICD-10: A00–R99)	1.3	(0.9 to 1.6)	0.2	(−0.2 to 0.5)	−0.3	(−0.7 to 0.1)
Cardiovascular disease (ICD-10: I00–99)	1.6	(0.8 to 2.4)	0.5	(−0.1 to 1.1)	−0.4	(−1.1 to 0.4)
Coronary heart disease (ICD-10: I20–25)	2.7	(1.0 to 4.4)	1.0	(−0.6 to 2.6)	1.0	(−0.1 to 2.2)
Stroke (ICD-10: I60–69)	1.3	(−0.2 to 2.8)	−0.3	(−1.4 to 0.8)	−0.3	(−1.5 to 0.9)
Respiratory disease (ICD-10: J00–99)	1.5	(0.7 to 2.3)	−0.3	(−1.3 to 0.8)	0.1	(−1.0 to 1.1)

CI, confidence interval; ICD, international classification of diseases; PM, particulate matter.

^a^Adjusted for PM_2.5_ at lag0-1, lag2, and lag3 simultaneously, ambient temperature at lag0-1, relative humidity at lag0-1, and influenza epidemics.

**Figure 1.**
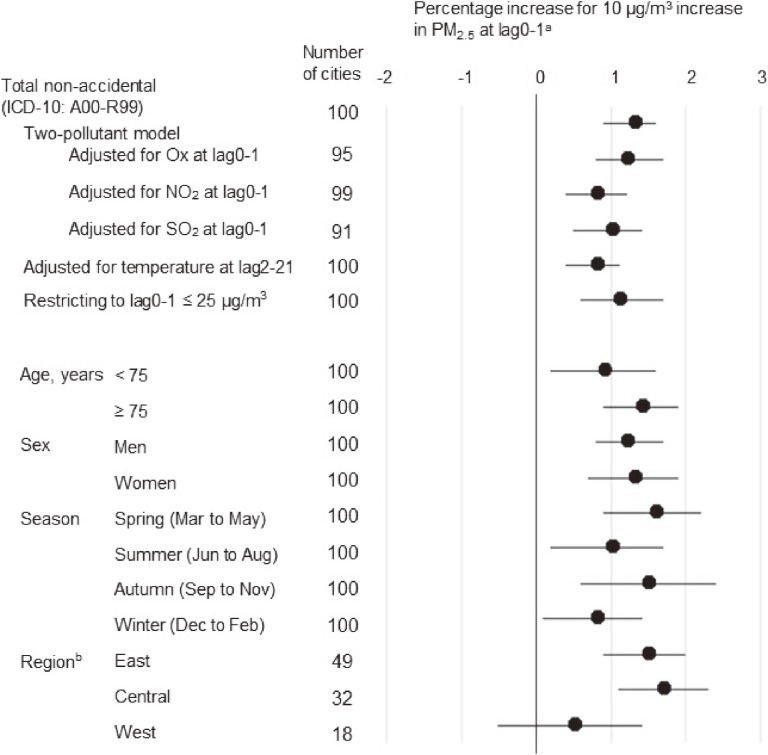
Sensitivity and stratified analyses of the association between PM_2.5_ exposure and total non-accidental mortality in 100 Japanese cities, April 2012 to March 2015 CI, confidence interval; ICD, international classification of diseases; NO_2_, nitrogen dioxide; Ox, photochemical oxidants; PM, particulate matter; SO_2_, sulphur dioxide ^a^Adjusted for PM_2.5_ at lag2 and lag3, ambient temperature at lag0-1, relative humidity at lag0-1, and influenza epidemics. ^b^The sum of the region was 99, because we did not include Naha, Okinawa, in West Japan due to geographical differences (see eTable 1).

We performed sensitivity and stratified analyses of the association between PM_2.5_ exposure and total non-accidental mortality (Figure 1). After adjustment for exposure to co-pollutants, or for temperature at long lag, the percentage increase point estimates were attenuated; however, the positive association between PM_2.5_ and mortality persisted. Even based on WHO guideline values, the total mortality increased by 1.1% (95% CI, 0.6–1.7%) with a 10 µg/m^3^ increase in PM_2.5_ at lag0-1. When we performed the stratified analyses, the point estimates of percentage increase tended to be slightly greater in adults aged 75 years or older and in spring and autumn. The PM_2.5_-mortality association varied somewhat by region: there was a positive association in East Japan (percentage increase, 1.5%; 95% CI, 0.9–2.0%) and in Central Japan (1.7%; 95% CI, 1.1–2.3%), but no clear positive association was found in West Japan (0.5%; 95% CI, −0.5 to 1.4%).

We analyzed the association between exposure to coarse PM and non-accidental mortality in 77 cities (eFigure 3), and the results of pooling the city-specific estimates are summarized in Table 4 and eTable 3. The coarse PM concentrations at lag0-1 were positively associated with an increased risk of total mortality (percentage increase per 10 µg/m^3^, 2.3%; 95% CI, 1.4–3.3%) without heterogeneity (I^2^ = 5.5%). After adjustment for exposure to PM_2.5_, though the pooled estimate of percentage increase was attenuated, the coarse PM-mortality association was positive (percentage increase, 1.4%; 95% CI, 0.2–2.6%). In the two-pollutant model, which included both coarse PM and PM_2.5_, the point estimates of percentage increase for the association of coarse PM with cardiovascular and respiratory mortality showed a positive direction but were not statistically significant (percentage increase for cardiovascular mortality, 1.3%; 95% CI, −0.9 to 3.5%; and percentage increase for respiratory mortality, 0.9%; 95% CI, −1.8 to 3.8%). With regard to PM_2.5_, adjustment for coarse PM did not substantially affect the pattern of the PM_2.5_-mortality association (Table 4).

**Table 4.** Pooled estimates for non-accidental mortality associated with PM_2.5_ and coarse PM in 77 Japanese cities, April 2012 to March 2015

Outcome	PM_2.5_		Coarse PM^a^	
	
	Percentage increase for 10 µg/m^3^ increase at lag0-1^b^	(95% CI)	Percentage increase for 10 µg/m^3^ increase at lag0-1^b^	(95% CI)
Single pollutant model				
Total non-accidental (ICD-10: A00–R99)	1.2	(0.8 to 1.7)	2.3	(1.4 to 3.3)
Cardiovascular disease (ICD-10: I00–99)	1.5	(0.6 to 2.4)	2.6	(0.7 to 4.4)
Coronary heart disease (ICD-10: I20–25)	1.8	(−0.2 to 3.9)	3.8	(0 to 7.7)
Stroke (ICD-10: I60–69)	1.0	(−0.6 to 2.7)	2.7	(−0.9 to 6.4)
Respiratory disease (ICD-10: J00–99)	1.5	(0.6 to 2.4)	1.4	(−0.8 to 3.7)
Two pollutant model^c^				
Total non-accidental (ICD-10: A00–R99)	1.2	(0.6 to 1.7)	1.4	(0.2 to 2.6)
Cardiovascular disease (ICD-10: I00–99)	1.4	(0.3 to 2.4)	1.3	(−0.9 to 3.5)
Coronary heart disease (ICD-10: I20–25)	2.5	(0.3 to 4.6)	2.5	(−1.7 to 6.7)
Stroke (ICD-10: I60–69)	0.6	(−1.0 to 2.3)	2.9	(−0.9 to 6.9)
Respiratory disease (ICD-10: J00–99)	2.1	(1.0 to 3.2)	0.9	(−1.8 to 3.8)

CI, confidence interval; ICD, international classification of diseases; PM, particulate matter.

^a^Concentrations of coarse PM were calculated by subtracting PM_2.5_ concentrations from those of suspended particulate matter.

^b^Adjusted for PM_2.5_ or coarse PM at lag2 and lag3, ambient temperature at lag0-1, relative humidity at lag0-1, and influenza epidemics.

^c^We included both PM_2.5_ and coarse PM in the model.

For coarse PM-mortality association, the elevated risk was more evident in West Japan than in East and Central Japan (eFigure 4).

## DISCUSSION

Under environmental conditions, in which the average (14.6 µg/m^3^) and 98th percentile (37.8 µg/m^3^) of daily mean PM_2.5_ concentration were similar to those of the Japan Air Quality Standard (annual mean ≤15 µg/m^3^, daily mean ≤35 µg/m^3^), an overall 10 µg/m^3^ increase in PM_2.5_ concentrations on the day of death and the previous day was associated with a 1.3% increase in total non-accidental mortality. This positive association was observed even when we restricted to days with PM_2.5_ concentrations at lag0-1 ≤25 µg/m^3^. The adverse effects of PM_2.5_ in this study were consistent with the previous, 20-cities study in Japan, which observed that a 10 µg/m^3^ increase on the previous day was associated with a 0.88% increase in non-accidental all-cause mortality for adults aged 65 years or older,^6^ though direct comparison of the effect size of the PM_2.5_-mortality association was difficult due to the difference in the number of cities, age category, and PM_2.5_ measurement method involved.

Our effect estimate of PM_2.5_ (a 1.3% increase in total mortality) tended to be higher than the estimates in the studies of other countries: 0.98% (95% CI, 0.75–1.22%) in 112 American cities from 1999–2005,^2^ 0.55% (95% CI, 0.27–0.84%) in 10 Mediterranean metropolitan areas from 2001–2010,^4^ and 0.8% (95% CI, 0.3–1.2%) in a nationwide Dutch study from 2008–2009,^16^ and 0.22% (95% CI, 0.15–0.28%) in 272 Chinese cities from 2013–2015.^3^ This discrepancy may be partly explained by differences in population structure. In Japan, the proportion of older population tends to be higher than in other countries.^17^ As we observed, the effect estimate of PM_2.5_ tended to be higher in those 75 years old or more, than in those less than 75 years old; and older individuals are likely more vulnerable to PM exposure.^18^ In addition, the difference may be influenced by regional differences in the composition and sources of PM_2.5_. Even in Japan, though a positive direction in the association between PM_2.5_ and mortality was observed in all three regions that were distinguished based on the composition and sources of PM_2.5_, the mortality effect of PM_2.5_ appeared to vary somewhat by region. In East Japan, domestic traffic-related pollution, as a source of elemental carbon (EC), made a major contribution to PM_2.5_ concentrations^15^^,^^19^; while sulfate (SO_4_^2−^) concentrations, influenced by transboundary pollution, were lower than in West Japan.^20^^,^^21^ Both EC and SO_4_^2−^ were associated with mortality, but the largest association per unit mass was observed for EC.^14^^,^^22^ Such regional differences in the composition and sources of PM_2.5_ may help interpret the apparent association in East Japan rather than in West Japan. Thus, a related health effect assessment, focused on the composition and sources of PM_2.5_,^23^ is required in the future. Among the possible reasons for seasonal difference, spring and autumn tend to witness an increase in outdoor activities, and decrease in air conditioner usage, meaning increased exposure to outdoor air pollution.

We found that exposure to PM_2.5_ was positively associated with both cardiovascular and respiratory mortality, and this was consistent with the results for the PM_2.5_-mortality association in past studies.^2^^–^^4^^,^^16^ With respect to cardiovascular disease, we found that PM_2.5_ exposure was associated with mortality due to coronary heart disease but not clearly associated with stroke mortality. The existence of acute cardiac effects due to PM_2.5_ exposure has been internationally accepted,^24^^,^^25^ and we believe that the supposition of a positive PM_2.5_-stroke association is not far from gaining consensus. Indeed, there is steadily increasing evidence suggesting a positive association between short-term exposure to PM_2.5_ and mortality due to stroke.^2^^,^^3^^,^^26^ In Tokyo, for example, PM_2.5_ exposure was associated with an elevated risk of elderly mortality related to stroke as well as coronary heart disease.^27^ In fact, the non-statistical elevated risk of stroke-related mortality we here observed may partially explain the fact that the acute fatality rate of coronary heart disease is higher than that of stroke.^28^

The health effects of coarse PM are still under investigation.^8^^,^^29^ In the American multi-city study,^2^ exposure to coarse PM, estimated as the difference between PM_10_ and PM_2.5_ (PM_10-2.5_), was associated with an increase in all-cause mortality; however, no such association was observed in the European multi-city study.^4^ In the present study, we used the difference between the respective concentrations of SPM and PM_2.5_ as a surrogate marker of coarse PM, and observed a 1.4% increase in total mortality with a 10 µg/m^3^ increase in coarse PM at lag0-1, after adjustment for PM_2.5_. This finding was similar to the Japanese single-city (ie, 23 wards of Tokyo) result for the association between coarse PM (defined as in our study) and all-cause mortality among adults aged 65 years or older between 2002 and 2013 (PM_2.5_-adjusted percentage increase, 1.6%; 95% CI, 1.1–2.2%).^27^ The clear association between coarse PM and mortality in West Japan might be partially related to the health effects of Asian dust originating in the deserts of China and Mongolia, which is mainly composed of coarse particles.^30^^–^^32^ There were more days of Asian dust in West Japan than in East and Central Japan.^33^ Our results did not show a clear association between coarse PM exposure and cause-specific mortality but did suggest a positive direction in the association. Coarse particles cause inflammation with deposition in the bronchial tubes, but, unlike fine particles, do not penetrate to the alveolus.^34^^,^^35^ Any significant conclusion regarding the health effects of coarse PM exposure in consideration of PM_2.5_ exposure would require further evidence.

The main strength of the present study is that it provides the first nationwide evidence of an association between mortality and short-term exposure to PM_2.5_ or coarse PM in Japan. Further, data for a large number of deaths were analyzed, and the effects of PM exposure on mortality were estimated with narrow CIs. Another strength of the study is its use of PM_2.5_ data measured using automated PM_2.5_ measuring equipment with measurement equivalence to a reference method. This enabled evaluation of the PM_2.5_-mortality association without allowance for systematic PM_2.5_ measurement error due to the measuring equipment. On the other hand, we should acknowledge some limitations in this study. There was random measurement error in the exposure assessment, and coarse PM (difference between SPM and PM_2.5_) was more likely to be susceptible to such error than PM_2.5_. Since this classical error appears to be unrelated to outcome, we must interpret the results of the association between coarse PM and mortality with allowance for an underestimate on the effect. As in past environmental epidemiologic studies investigating acute health effects of air pollution, another limitation of the study was the inevitability of exposure misclassification, giving that the results were based on the use of pollutant data measured at a single monitoring station in each city. It was difficult to estimate the biasing effect of a combination of this Berkson error and classical error on our estimates of the PM-mortality association.^36^

In conclusion, we observed that short-term exposure to PM_2.5_ had adverse effects on total non-accidental, cardiovascular, and respiratory mortality in Japan. Coarse PM exposure also increased the risk of total mortality. Our findings suggest that we must continue monitoring for exposure to PM, including the coarse component, and continue assessing the health effects of such exposure.

## APPENDIX A. SUPPLEMENTARY DATA

eTable 1. Descriptive data on the study period, total non-accidental death, and environmental factors for 100 Japanese cities

eTable 2. Pearson’s correlation coefficients among daily mean pollutant concentrations

eTable 3. Pooled estimates for the association between coarse PM exposure and mortality in 77 Japanese cities, April 2012 to March 2015

eFigure 1. Location of monitoring stations in 100 Japanese cities

eFigure 2. City-specific odds ratios (ORs) of total non-accidental death with a 10 µg/m^3^ increase in PM_2.5_ concentrations at lag0-1

eFigure 3. City-specific odds ratios (ORs) of total non-accidental death with a 10 µg/m^3^ increase in coarse particulate matter concentrations at lag0-1

eFigure 4. Sensitivity and stratified analyses of the association between coarse PM exposure and total non-accidental mortality
