# Supplementary material for: Japanese Nationwide Study on the Association Between Short-term Exposure to Particulate Matter and Mortality
Source: J Epidemiol. 2019 Dec 5;29(12):471–7. doi: 10.2188/jea.JE20180122 (PMC6859079; doi:10.2188/jea.JE20180122)
Supplement: Supplementary file 1 [file je-29-471-s001.pdf]

eTable 1. Descriptive data on the study period, total non-accidental death, and environmental factors for 100 Japanese cities

				2015 Population Census			Total non-accidental death	$PM_{2.5}$ (μg/m^3)							Coarse PM (μg/m^3)		Ox (ppb)		NO_2 (ppb)		SO_2 (ppb)		Ambient temperature (℃)		Relative humidity (%)	
	Prefecture	City	Region	Population	Area (km^2)	Study period	(ICD-10: A00-R99)	Days	Frequency of days with daily mean > 25 μg/m^3	Mean	SD	25th per-centile	Median	75th per-centile	Mean	SD	Mean	SD	Mean	SD	Mean	SD	Mean	SD	Mean	SD
1	Hokkaido	Sapporo	East	1952356	1121.3	4/12−3/15	39380	1091	2.2	8.0	6.1	4.2	6.8	10.5	4.0	2.6	41.5	11.4	10.1	7.6	0.8	1.0	9.6	9.8	70	11
2		Asahikawa	East	339605	747.7	4/12−3/15	10612	1088	7.2	13.3	8.6	8.1	11.3	15.6					11.7	10.4	0.8	0.8	7.6	11.0	76	9
3		Hakodate	East	265979	677.9	4/12−3/15	9406	1083	4.6	12.6	6.7	8.1	11.5	15.3					11.6	6.2	1.4	0.9	9.8	9.4	75	9
4	Aomori	Aomori	East	287648	824.6	4/13−3/15	6131	724	5.5	11.9	7.1	7.5	10.3	14.4	4.2	4.8			7.8	5.2			10.9	9.4	76	10
5		Hachinohe	East	231257	305.5	4/12−3/15	6823	1087	5.9	12.3	7.4	7.0	10.7	15.6					8.5	4.2	2.8	1.9	10.6	9.0	76	12
6	Iwate	Morioka	East	297631	886.5	4/12−3/15	7424	1080	5.4	12.1	6.8	7.2	10.8	15.7			37.1	11.3	9.0	5.8	0.4	0.4	11.0	9.7	75	11
7	Miyagi	Sendai	East	1082159	786.3	4/12−3/15	21332	1081	5.6	11.7	7.6	6.3	10.3	15.7			38.3	11.5	10.7	5.4	0.5	0.5	13.0	8.6	71	13
8	Akita	Akita	East	315814	906.1	4/12−3/15	9295	1081	8.1	13.6	8.1	8.0	11.5	16.9	6.7	6.2	43.9	10.2	4.7	2.7	1.1	0.7	12.3	9.4	73	10
9	Yamagata	Yamagata	East	253832	381.3	4/12−3/15	7202	1088	6.7	12.5	8.0	6.8	10.8	16.6	2.5	3.6	41.1	12.5	10.3	6.6	1.2	0.8	12.1	9.7	74	11
10	Fukushima	Fukushima	East	294247	767.7	4/14−3/15	2801	363	5.8	11.3	6.9	6.0	10.4	14.9	7.9	2.5	38.9	12.2	9.6	5.8	0.7	0.5	13.5	9.1	69	12
11		Koriyama	East	335444	757.2	4/13−3/15	5551	726	6.3	12.0	7.4	6.7	10.5	15.1	3.7	2.5	41.8	12.6	9.6	4.9	1.3	0.5	12.0	8.9	73	13
12		Iwaki	East	350237	1232.0	4/13−3/15	7385	722	8.2	12.3	8.1	6.5	10.3	16.5	8.8	4.4	40.0	12.2	8.8	4.9	2.7	2.5	13.8	7.6	72	15
13	Ibaraki	Mito	East	270783	217.3	4/12−3/15	6624	1084	5.1	12.2	7.0	7.1	10.6	15.5	4.2	4.7	43.2	13.6	7.6	3.2	0.6	0.4	14.3	8.3	73	14
14		Tsukuba-Tsuchiura	East	367767	406.6	4/12−3/15	7804	1038	12.9	15.9	9.0	9.8	14.2	19.8	8.6	4.4	41.0	15.0	11.0	6.2	0.6	0.5	14.5	8.6	72	14
15	Tochigi	Utsunomiya	East	518594	416.9	4/13−3/15	7985	729	8.0	13.5	7.5	8.0	12.4	17.3	3.9	3.9	46.0	17.1	10.3	5.3			14.4	8.8	67	14
16	Gunma	Maebashi	East	336154	311.6	4/12−3/15	9343	1065	14.0	15.3	9.1	8.3	14.4	20.8			49.1	16.8	8.0	4.0	1.5	0.7	15.2	8.8	60	14
17		Takasaki	East	370884	459.2	4/14−3/15	3360	360	14.4	15.7	9.8	8.8	13.5	20.4	6.1	6.0					1.1	0.4	15.2	8.8	60	14
18		Ota	East	219807	175.5	4/13−3/15	3453	701	11.1	15.0	8.4	8.7	13.6	19.7			47.2	18.4	12.4	6.6	1.9	0.7	15.2	8.8	60	14
19	Saitama	Saitama	East	1263979	217.4	4/13−3/15	16858	726	9.9	14.4	8.3	8.4	12.9	18.6	7.1	7.0	42.9	17.1	15.7	7.4	0.9	0.6	15.6	8.7	63	16
20		Tokorozawa	East	340386	72.1	4/12−3/15	6873	1084	7.2	14.1	7.4	8.8	13.2	17.8			44.9	17.4	13.6	6.0	0.6	0.5	15.6	8.7	63	16
21		Kawaguchi	East	578112	62.0	4/13−3/15	8140	722	13.3	14.9	9.6	8.1	12.4	19.3	4.6	6.4	41.2	16.8	20.7	9.1	2.0	1.2	15.6	8.7	63	16
22		Kawagoe	East	350745	109.1	4/14−3/15	2672	361	10.2	14.8	8.1	8.4	13.6	19.9			44.2	17.5	14.6	6.5	1.2	0.7	15.6	8.7	63	16
23		Koshigaya	East	337498	60.2	4/13−3/15	4662	721	11.0	15.0	8.4	9.0	13.2	19.4	4.1	4.9	43.4	18.2	16.0	8.1	1.5	0.8	15.6	8.7	63	16
24	Chiba	Chiba	East	971882	271.8	4/12−3/15	20053	1089	14.0	15.2	10.5	7.8	12.6	20.0	10.2	5.9	40.5	15.2	11.9	6.4	1.8	1.1	16.4	7.9	65	16
25		Ichikawa	East	481732	57.5	4/12−3/15	8722	1028	14.0	15.2	9.9	8.5	13.4	19.5	5.7	5.5	41.3	16.7	16.6	8.9	1.2	1.0	16.4	7.9	65	16
26		Funabashi	East	622890	85.6	4/12−3/15	11970	1081	8.1	13.0	8.3	7.0	11.2	17.1			40.0	15.3	13.5	7.6	1.6	1.1	16.4	7.9	65	16
27		Matsudo	East	483480	61.4	4/12−3/15	10127	1084	9.4	13.7	8.8	7.5	11.8	17.8			37.1	15.0	17.9	8.3	1.1	0.9	16.4	7.9	65	16
28		Kashiwa	East	413954	114.7	4/12−3/15	7722	1060	8.4	13.5	8.2	7.7	12.0	17.7	4.3	5.2	44.1	16.6	14.2	7.8	3.5	1.2	16.4	7.9	65	16
29		Ichihara	East	274656	368.2	4/12−3/15	6364	1089	12.2	15.3	8.5	9.2	13.0	19.0	3.3	4.9	40.8	15.5	12.3	7.2	3.9	1.5	16.4	7.9	65	16
30	Tokyo	Tokyo's 23 wards	East	9272740	626.7	4/12−3/15	199866	1073	15.9	17.0	9.0	10.5	15.4	21.2	3.8	3.7	35.4	14.6	22.9	9.3	2.0	1.1	16.8	8.2	62	16
31		Hachioji	East	577513	186.4	4/12−3/15	12788	1093	5.7	12.8	6.7	8.0	11.5	15.9			41.6	17.9	16.3	6.3	1.4	0.4	16.8	8.2	62	16
32		Machida	East	432348	71.8	4/12−3/15	8924	1073	9.6	14.7	7.8	9.2	13.3	18.5	5.2	4.3	45.4	18.9	14.1	6.5	1.7	0.6	16.8	8.2	62	16
33		Fuchu	East	260274	29.4	4/13−3/15	3318	710	11.0	15.0	8.1	9.0	13.7	18.8	5.4	4.2	44.3	19.1	17.3	7.5			16.8	8.2	62	16
34		Chofu	East	229061	21.6	4/13−3/15	2647	663	6.9	12.9	7.0	7.8	11.8	16.2	6.8	4.7	42.0	18.2	15.3	7.1			16.8	8.2	62	16
35		Nishitokyo	East	200012	15.8	4/13−3/15	2564	718	11.6	15.5	8.7	9.2	14.1	19.6	4.9	4.3	47.0	19.1	14.2	7.2			16.8	8.2	62	16
36	Kanagawa	Yokohama	East	3724844	437.5	4/12−3/15	78088	1083	11.3	14.7	8.9	8.4	12.8	18.5	10.7	6.3	38.2	15.7	18.2	8.4	4.0	3.1	16.4	7.8	66	16
37		Kawasaki	East	1475213	143.0	4/12−3/15	25624	1081	7.7	13.6	7.7	7.9	12.1	17.4	5.1	5.0	41.6	17.4	18.6	8.9	1.4	1.1	16.4	7.8	66	16
38		Yokosuka	East	406586	100.8	4/13−3/15	7990	708	9.2	14.7	8.4	8.8	12.8	18.3	6.5	6.1	43.6	16.3	14.8	8.2	2.6	1.4	16.4	7.8	66	16
39		Sagamihara	East	173612	253.7	4/12−3/15	14523	1083	9.4	13.8	8.9	7.5	12.0	18.1	7.1	4.1	42.3	17.2	16.0	6.7	2.9	1.1	16.4	7.8	66	16
40		Hiratsuka	East	173612	253.7	4/13−3/15	3942	703	8.5	14.7	7.6	9.8	13.3	18.0	6.9	4.6	41.4	17.2	14.2	7.2	0.9	0.7	16.4	7.8	66	16
41		Fujisawa	East	423894	69.6	4/14−3/15	2857	357	7.3	13.6	7.5	8.4	12.2	17.3	6.0	4.0	44.4	17.1	14.5	8.1	1.1	0.7	16.4	7.8	66	16
42		Yamato	East	232922	27.1	4/12−3/15	4612	1059	8.4	14.3	7.6	8.8	12.8	18.3	7.1	3.3	36.1	15.4	19.4	7.3	2.4	0.9	16.4	7.8	66	16
43		Atsugi	East	225714	93.8	4/14−3/15	1434	362	4.4	11.6	6.9	6.9	10.4	14.9	13.7	6.5	40.6	17.6	16.1	7.4	1.3	0.6	16.4	7.8	66	16
44	Niigata	Niigata	East	810157	726.5	4/12−3/15	21735	1074	7.7	12.7	8.0	7.3	11.1	16.0	8.3	3.8	44.8	11.3	8.6	4.2	1.5	0.9	14.0	9.0	72	10
45	Toyama	Toyama	Central	418686	1241.8	4/12−3/15	11842	1095	5.9	10.7	7.7	5.6	9.2	14.3	4.1	3.8	46.2	12.2	8.3	4.1	0.9	0.5	14.5	9.2	74	12
46	Ishikawa	Kanazawa	Central	465699	468.6	4/13−3/15	7601	719	7.9	12.7	7.8	7.5	10.9	15.7	4.7	4.4	44.5	13.4	9.7	4.3	1.4	1.6	15.1	9.0	70	10
47	Fukui	Fukui	Central	265904	536.4	4/12−3/15	7199	1048	12.0	15.7	8.9	9.5	14.2	19.8	4.6	5.3	43.7	15.1	8.2	4.0	1.2	0.8	14.9	9.2	74	11
48	Nagano	Nagano	East	377598	834.8	4/12−3/15	10529	1063	4.8	11.7	7.1	6.5	10.6	15.7	3.6	3.7	44.9	14.4	5.8	4.6	3.5	1.6	12.3	9.8	72	11
49		Matsumoto	East	243293	978.5	4/12−3/15	6348	1071	3.1	11.0	6.5	6.0	9.9	15.0	4.9	4.8	40.3	12.8	9.4	6.2	3.4	0.9	12.3	9.6	66	12
50	Gifu	Gifu	Central	406735	203.6	4/12−3/15	11140	1052	7.3	13.1	8.1	7.3	11.9	17.1			45.3	15.3	9.3	3.9	0.7	0.7	16.2	8.9	65	13
51	Shizuoka	Shizuoka	East	704989	1411.9	4/13−3/15	9871	724	7.0	12.9	8.0	7.4	10.8	16.2	8.2	7.1	43.5	14.7	10.1	4.0	1.6	0.7	16.9	7.6	66	14
52		Hamamatsu	East	797980	1558.1	4/12−3/15	20486	1081	8.7	13.3	8.3	7.6	12.0	17.2	4.1	4.4	40.4	15.5	6.8	4.1	4.0	1.8	16.7	7.9	68	15
53		Fuji	East	248399	245.0	4/12−3/15	5278	1083	7.0	13.7	7.0	8.8	12.4	16.8	15.3	7.7	39.8	15.9	19.6	6.4	2.8	1.2	16.4	7.9	68	10
54	Aichi	Nagoya	Central	2295638	326.5	4/12−3/15	53545	1074	15.2	16.3	9.2	9.7	14.9	21.0	6.4	4.5	43.3	15.5	15.4	7.8	1.5	0.8	16.2	8.8	65	13
55		Toyohashi	Central	374765	261.9	4/12−3/15	8717	927	9.2	13.6	8.0	7.6	11.9	17.0			46.9	15.7	7.9	4.9			16.2	8.8	65	13
56		Ichinomiya	Central	380868	113.8	4/12−3/15	9034	1073	11.4	15.4	8.2	9.5	14.3	19.3	7.6	6.9	42.1	15.0	13.7	5.8	1.4	0.9	16.2	8.8	65	13
57		Toyota	Central	422542	918.3	4/13−3/15	5098	726	6.5	11.7	7.8	6.0	10.2	15.4	6.8	4.1	47.3	17.1	7.6	4.2			16.2	8.8	65	13
58	Mie	Tsu	Central	279886	711.1	4/12−3/15	7870	1085	8.3	13.4	8.0	7.5	12.0	17.2	12.4	5.8	46.6	14.6	7.4	4.5	1.6	0.6	16.3	8.4	67	13
59		Yokkaichi	Central	311031	206.4	4/14−3/15	2626	363	9.6	14.1	7.9	8.3	12.5	18.0	5.5	6.0	43.9	13.4	12.2	6.3	0.8	0.8	15.1	8.4	73	12
60	Shiga	Otsu	Central	340973	464.5	4/13−3/15	5108	722	8.3	13.1	8.1	7.0	11.3	16.7	5.5	5.2	45.7	13.5	7.7	4.2			15.1	8.8	72	9
61	Kyoto	Kyoto	Central	1475183	827.8	4/12−3/15	37931	1072	8.2	14.0	7.7	8.4	12.3	17.9			43.0	14.7	12.5	5.9	3.7	1.1	16.2	8.9	65	10
62	Osaka	Osaka	Central	2691185	225.2	4/12−3/15	73116	1084	17.8	17.5	9.4	10.4	15.5	22.0	9.5	4.6	36.6	14.5	23.9	9.5	4.3	2.1	16.9	8.5	63	11
63		Sakai	Central	839310	149.8	4/13−3/15	14097	726	10.3	14.2	8.7	7.6	12.8	19.0	6.9	4.8	46.4	17.3	15.0	7.3	5.5	2.3	16.9	8.5	63	11
64		Toyonaka	Central	395479	36.4	4/13−3/15	5867	714	13.5	16.4	8.3	10.2	14.9	20.9	10.0	11.2	42.2	14.6	16.7	8.2	4.2	1.9	16.9	8.5	63	11
65		Suita	Central	374468	36.1	7/12−3/15	6576	986	9.7	14.8	7.8	9.0	12.9	18.9			45.0	16.2	13.5	7.2	3.0	1.6	16.9	8.5	63	11
66		Higashiosaka	Central	502784	61.8	4/13−3/15	8475	638	11.8	14.4	9.0	7.8	12.7	19.2	7.1	4.3	43.9	16.2	16.9	8.7	5.8	2.2	16.9	8.5	63	11
67		Yao	Central	268800	41.7	4/12−3/15	7184	1082	15.3	16.2	9.4	9.1	14.4	20.9	5.5	3.6	44.1	16.7	16.5	8.0	2.1	1.0	16.9	8.5	63	11
68		Hirakata	Central	404152	65.1	4/14−3/15	2936	363	6.6	13.4	6.9	8.1	12.3	16.7	3.6	3.7	44.8	16.9	12.7	6.4	0.6	0.7	16.9	8.5	63	11
69		Ibaraki	Central	280033	76.5	4/14−3/15	1919	362	6.6	13.3	7.0	8.3	12.1	17.0	4.9	4.3	40.1	15.2	16.1	7.6	2.1	1.1	16.9	8.5	63	11
70		Neyagawa	Central	237518	24.7	4/12−3/15	5505	1075	9.9	13.9	8.5	7.5	12.6	18.0	8.3	5.3	43.2	16.5	16.2	7.7			16.9	8.5	63	11
71		Takatsuki	Central	351829	105.3	4/13−3/15	5572	697	11.1	14.7	7.9	8.7	13.3	19.3	6.4	5.1	44.0	16.5	13.5	7.3	1.9	0.8	16.9	8.5	63	11
72	Hyogo	Kobe	Central	1537272	557.0	4/13−3/15	26460	726	12.4	15.4	8.8	8.8	13.8	20.2	7.2	6.5	38.8	14.1	16.2	8.6	3.5	1.9	17.0	8.4	63	11
73		Himeji	Central	535664	534.5	4/13−3/15	9157	719	12.7	15.5	8.6	9.0	14.0	19.8			41.3	15.6	9.6	4.1	0.9	0.7	15.5	8.7	71	10
74		Akashi	Central	293409	49.4	4/12−3/15	7033	1055	15.1	16.4	9.1	9.8	14.5	20.8	7.0	9.6	41.1	13.9	15.9	7.8	2.2	1.9	15.5	8.7	71	10
75		Kakogawa	Central	267435	138.5	4/12−3/15	6113	1080	21.1	18.3	10.0	11.4	16.5	23.5	4.4	6.0	44.4	15.0	13.0	6.2	1.5	1.3	15.5	8.7	71	10
76		Amagasaki	Central	452563	50.7	4/14−3/15	4124	363	13.0	16.7	8.0	10.6	15.4	20.7			38.7	14.8	18.2	8.3	1.7	1.1	17.0	8.4	63	11
77		Takarazuka	Central	224903	101.8	4/14−3/15	1753	361	5.5	12.3	6.8	7.3	11.3	15.7			43.8	15.3	14.2	7.3	0.8	0.9	17.0	8.4	63	11
78	Nara	Nara	Central	360310	276.9	4/12−3/15	8880	1079	9.6	13.8	8.4	7.8	12.1	17.8	3.3	3.3	42.1	14.7	9.8	5.0	3.7	1.3	15.1	8.7	73	11
79	Wakayama	Wakayama	Central	364154	208.8	4/12−3/15	11628	1087	12.3	15.8	8.3	9.9	14.4	19.9	7.4	6.1	44.2	15.2	9.0	3.8	2.7	1.2	16.9	8.3	66	11
80	Okayama	Okayama	West	364154	208.8	4/12−3/15	17053	1089	21.7	18.6	9.6	11.7	16.6	23.7	5.9	6.4	42.9	16.1	11.8	5.1	5.1	1.6	16.3	8.8	65	11
81		Kurashiki	West	477118	355.6	4/12−3/15	12055	1085	18.3	17.3	9.4	10.3	15.8	22.5			45.6	15.5	13.8	6.1	4.7	1.6	16.3	8.8	65	11
82	Hiroshima	Hiroshima	West	1194034	906.5	4/12−3/15	25734	1089	21.8	19.0	8.7	12.4	17.8	24.1	11.1	4.9	43.9	16.5	13.6	6.1	1.1	0.9	16.5	8.6	67	11
83		Fukuyama	West	464811	518.1	4/12−3/15	12191	1090	17.4	16.4	9.6	9.2	14.9	22.0	7.8	5.1	41.9	15.1	16.2	6.7	1.7	1.9	15.7	8.8	72	10
84		Kure	West	228552	352.8	4/14−3/15	2866	365	20.0	18.0	9.1	11.5	16.3	23.3	4.7	4.1	44.9	14.2	10.4	5.0	2.0	1.7	16.4	8.1	66	11
85	Yamaguchi	Shimonoseki	West	268517	715.9	4/12−3/15	9641	1093	18.6	17.5	9.7	10.5	15.8	22.3	4.8	5.6	45.3	14.6	12.7	7.3	2.5	1.5	17.0	7.7	68	12
86	Tokushima	Tokushima	Central	258554	191.3	4/12−3/15	7438	1061	10.7	14.7	8.6	8.6	13.0	19.2			42.0	15.3	9.5	4.3	1.4	0.8	16.7	8.1	68	12
87	Kagawa	Takamatsu	West	420748	375.4	4/12−3/15	11025	1080	21.4	18.5	10.1	11.2	16.6	23.4	5.5	5.4	38.9	13.2	15.0	8.3	6.4	3.4	16.7	8.6	66	12
88	Ehime	Matsuyama	West	514865	429.4	4/12−3/15	13575	1092	17.0	17.1	8.8	10.4	15.8	21.7	4.1	5.8	40.0	13.1	10.8	4.8	3.0	2.2	16.7	8.2	67	12
89	Kochi	Kochi	Central	337190	309.0	4/12−3/15	9480	1047	12.0	15.2	8.0	9.5	13.4	19.3			42.7	13.8	5.7	3.3	3.5	1.2	17.1	7.9	69	13
90	Fukuoka	Fukuoka	West	1538681	343.4	4/12−3/15	28192	1090	21.7	18.9	9.7	11.5	17.2	23.8	6.5	5.4	39.7	14.1	16.9	5.5	2.4	1.4	17.4	8.0	67	13
91		Kitakyushu	West	961286	492.0	4/12−3/15	28585	1092	28.7	21.0	10.3	13.6	18.7	26.1	6.0	7.1	39.9	14.5	15.8	6.9	2.1	1.4	17.4	8.0	67	13
92		Kurume	West	304552	230.0	4/13−3/15	5313	721	25.4	19.1	10.7	11.2	17.2	25.2	7.6	6.8	43.0	16.4	10.7	4.7	1.2	1.1	17.4	8.0	67	13
93	Saga	Saga	West	236372	431.8	4/12−3/15	6807	1081	14.5	15.4	9.4	8.4	13.4	20.5	6.9	4.0	44.1	14.8	8.3	4.3	1.9	1.1	16.9	8.3	71	11
94	Nagasaki	Nagasaki	West	429508	405.9	4/14−3/15	4452	363	17.1	17.8	9.1	11.2	16.4	22.9			43.3	15.0	6.9	3.4	1.8	1.1	17.3	7.7	72	11
95		Sasebo	West	255439	426.1	4/12−3/15	8271	1059	26.0	20.0	10.4	12.3	17.7	25.5	2.2	3.6	39.9	13.8	9.9	4.5	1.4	0.9	17.2	7.8	68	12
96	Kumamoto	Kumamoto	West	740822	390.3	4/13−3/15	11525	673	25.1	19.1	10.3	11.0	17.9	25.1	5.5	4.2	44.6	15.4	7.0	4.3	3.3	2.9	17.0	8.3	70	11
97	Oita	Oita	West	478146	502.4	4/12−3/15	10188	1064	15.7	16.2	9.3	9.0	14.8	21.4	3.0	4.8	42.5	14.7	7.3	2.9	2.6	1.5	16.6	7.9	69	12
98	Miyazaki	Miyazaki	West	401138	643.7	5/13−3/15	6122	682	10.6	13.6	8.7	7.6	12.0	18.1	13.6	8.1	30.8	12.6	5.0	2.8	4.1	2.5	17.6	7.7	74	13
99	Kagoshima	Kagoshima	West	599814	547.6	4/12−3/15	15178	1036	19.5	17.9	9.0	11.4	16.5	23.0	4.9	6.3	40.4	12.7	7.1	4.0	2.5	5.0	18.6	7.5	72	11
100	Okinawa	Naha		319435	39.6	4/14−3/15	2200	352	5.4	10.9	6.7	6.3	8.9	13.1	11.1	6.0	32.3	16.3	6.1	4.1	1.3	2.2	23.1	4.8	73	11

ICD, international classification of diseases; NO_2, nitrogen dioxide; Ox, photochemical oxidants; PM, particulate matter; SD, standard deviation; SO_2, sulphur dioxide.

eTable 2. Pearson's correlation coefficients among daily mean pollutant concentrations

	$PM_{2.5}$ (μg/m^3)	Coarse PM (μg/m^3)[a]	Ox (ppb)[b]	NO_2 (ppb)	SO_2 (ppb)
$PM_{2.5,}$ μg/m^3	1				
Coarse PM, μg/m^3[a]	0.33	1			
Ox, ppb[b]	0.37	0.09	1		
$NO_{2,}$ ppb	0.47	0.05	-0.20	1	
$SO_{2,}$ ppb	0.42	0.24	0.26	0.35	1

NO_2, nitrogen dioxide; Ox, photochemical oxidants; PM, particulate matter; SO_2, sulphur dioxide.

[a]Concentrations of coarse PM were calculated by subtracting $PM_{2.5}$ concentrations from those of suspended particulate matter.

[b]Daily maximum 8-h mean concentrations.

eTable 3. Pooled estimates for the association between coarse PM exposure and mortality in 77 Japanese cities, April 2012 to March 2015

	Lag0-1		Lag2		Lag3	
	Percentage increase for 10 μ g/m^3 increase in coarse PM[a,b]	(95% CI)	Percentage increase for 10 μ g/m^3 increase in coarse PM[a,b]	(95% CI)	Percentage increase for 10 μ g/m^3 increase in coarse PM[a,b]	(95% CI)
Total non-accidental (ICD-10: A00-R99)	2.3	(1.4 to 3.3)	-1.4	(-2.2 to -0.7)	-0.9	(-1.6 to -0.2)
Cardiovascular disease (ICD-10: I00-99)	2.6	(0.7 to 4.4)	-0.9	(-2.4 to 0.5)	-0.5	(-1.8 to 0.8)
Coronary heart disease (ICD-10: I20-25)	3.8	(0 to 7.7)	0.2	(-3.7 to 4.2)	1.2	(-2.2 to 4.6)
Stroke (ICD-10: I60-69)	2.7	(-0.9 to 6.4)	-2.2	(-5.2 to 0.8)	-1.2	(-3.9 to 1.5)
Respiratory disease (ICD-10: J00-99)	1.4	(-0.8 to 3.7)	-2.3	(-4.5 to 0)	-0.8	(-2.7 to 1.1)

CI, confidence interval; ICD, international classification of diseases; PM, particulate matter.

[a]Concentrations of coarse PM were calculated by subtracting $PM_{2.5}$ concentrations from those of suspended particulate matter.

[b]Adjusted for coarse PM at lag0-1, lag2, and lag3 simultaneously, ambient temperature at lag0-1, relative humidity at lag0-1, and influenza epidemics.

eFigure 1. Location of monitoring stations in 100 Japanese cities

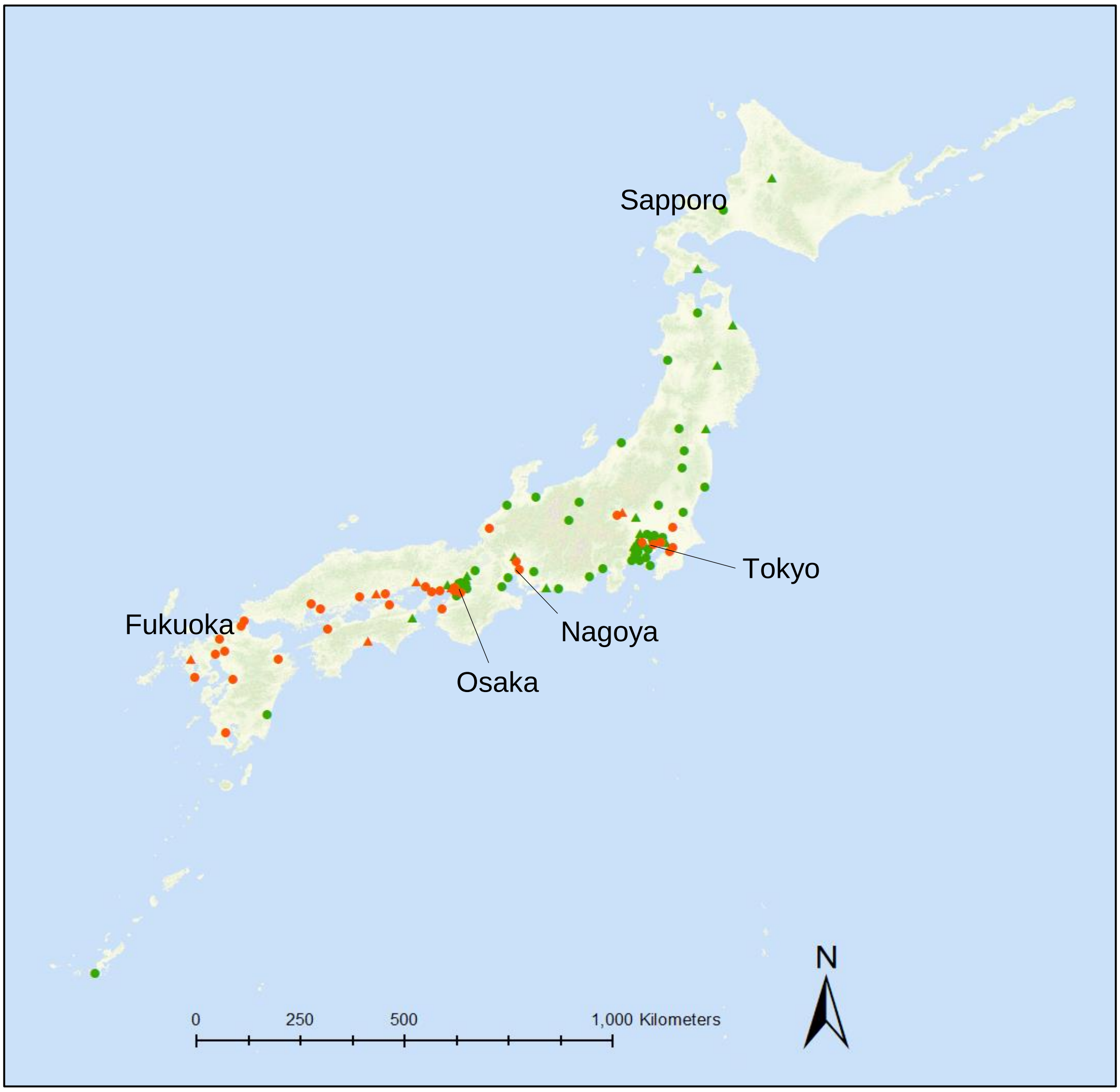

There are 77 cities included in the analysis of the coarse PM-mortality association (circle), and 23 cities not included (triangle). Green color means the average daily mean $PM_{2.5}$ concentration was ≤ 15 μg/m^3, and orange color means that was > 15 μg/m^3.

eFigure 2. City-specific odds ratios (ORs) of total non-accidental death with a 10 μg/m^3 increase in $PM_{2.5}$ concentrations at lag0-1

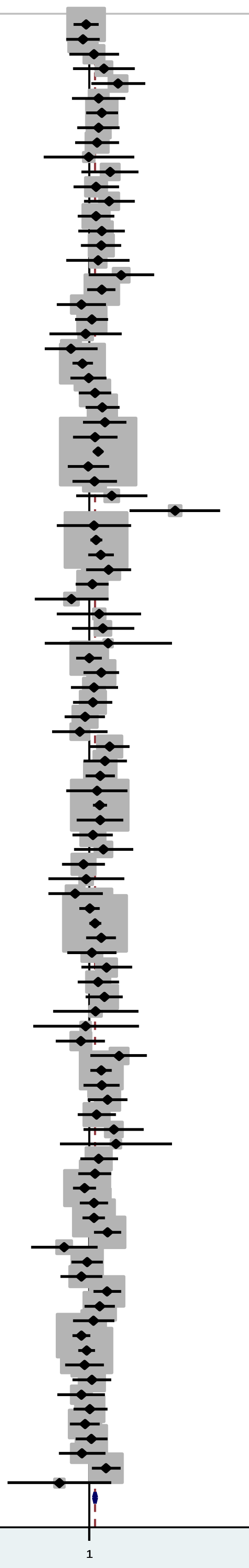

code	city	OR (95% CI)	% Weight
1	Sapporo	0.99 (0.97, 1.02)	1.69
2	Asahikawa	0.99 (0.95, 1.02)	1.03
3	Hakodate	1.01 (0.96, 1.07)	0.49
4	Aomori	1.03 (0.97, 1.10)	0.32
5	Hachinohe	1.06 (1.00, 1.13)	0.42
6	Morioka	1.02 (0.96, 1.08)	0.43
7	Sendai	1.03 (0.99, 1.06)	1.14
8	Akita	1.02 (0.98, 1.07)	0.66
9	Yamagata	1.02 (0.97, 1.07)	0.62
10	Fukushima	1.00 (0.91, 1.10)	0.15
11	Koriyama	1.05 (0.98, 1.11)	0.38
12	Iwaki	1.01 (0.97, 1.07)	0.58
13	Mito	1.04 (0.99, 1.10)	0.47
14	Tsukuba-Tsuchiura	1.01 (0.98, 1.05)	0.89
15	Utsunomiya	1.03 (0.98, 1.08)	0.56
16	Maebashi	1.03 (0.98, 1.07)	0.73
17	Takasaki	1.02 (0.95, 1.09)	0.31
18	Ota	1.07 (1.00, 1.15)	0.29
19	Saitama	1.03 (1.00, 1.06)	1.40
20	Tokorozawa	0.98 (0.93, 1.04)	0.50
21	Kawaguchi	1.01 (0.97, 1.04)	1.04
22	Kawagoe	0.99 (0.92, 1.07)	0.24
23	Koshigaya	0.96 (0.91, 1.02)	0.44
24	Chiba	0.99 (0.96, 1.01)	2.43
25	Ichikawa	1.00 (0.96, 1.04)	0.90
26	Funabashi	1.01 (0.98, 1.05)	1.08
27	Matsudo	1.03 (0.99, 1.07)	0.98
28	Kashiwa	1.03 (0.99, 1.08)	0.64
29	Ichihara	1.01 (0.97, 1.06)	0.60
30	Tokyo's 23 wards	1.02 (1.01, 1.03)	7.18
31	Hachioji	1.00 (0.95, 1.04)	0.69
32	Machida	1.01 (0.96, 1.06)	0.61
33	Fuchu	1.05 (0.97, 1.13)	0.25
34	Chofu	1.20 (1.09, 1.32)	0.15
35	Nishitokyo	1.01 (0.93, 1.09)	0.23
36	Yokohama	1.01 (1.00, 1.03)	4.83
37	Kawasaki	1.02 (1.00, 1.05)	1.67
38	Yokosuka	1.04 (0.99, 1.09)	0.60
39	Sagamihara	1.01 (0.97, 1.04)	1.06
40	Hiratsuka	0.96 (0.89, 1.04)	0.23
41	Fujisawa	1.02 (0.93, 1.12)	0.18
42	Yamato	1.03 (0.96, 1.10)	0.32
43	Atsugi	1.04 (0.91, 1.19)	0.08
44	Niigata	1.00 (0.97, 1.03)	1.65
45	Toyama	1.03 (0.99, 1.07)	0.93
46	Kanazawa	1.01 (0.96, 1.06)	0.55
47	Fukui	1.01 (0.97, 1.05)	0.77
48	Nagano	0.99 (0.95, 1.03)	0.74
49	Matsumoto	0.98 (0.92, 1.04)	0.40
50	Gifu	1.04 (1.00, 1.09)	0.74
51	Shizuoka	1.03 (0.99, 1.08)	0.64
52	Hamamatsu	1.02 (0.99, 1.06)	1.29
53	Fuji	1.02 (0.95, 1.09)	0.33
54	Nagoya	1.02 (1.01, 1.04)	4.11
55	Toyohashi	1.02 (0.97, 1.08)	0.56
56	Ichinomiya	1.01 (0.96, 1.05)	0.73
57	Toyota	1.03 (0.97, 1.10)	0.35
58	Tsu	0.99 (0.94, 1.03)	0.66
59	Yokkaichi	0.99 (0.92, 1.08)	0.22
60	Otsu	0.97 (0.92, 1.03)	0.41
61	Kyoto	1.00 (0.98, 1.02)	2.39
62	Osaka	1.01 (1.00, 1.03)	5.03
63	Sakai	1.03 (0.99, 1.06)	1.29
64	Toyonaka	1.01 (0.95, 1.06)	0.50
65	Suita	1.04 (0.98, 1.10)	0.48
66	Higashiosaka	1.02 (0.98, 1.07)	0.70
67	Yao	1.03 (0.99, 1.08)	0.86
68	Hirakata	1.01 (0.93, 1.11)	0.17
69	Ibaraki	0.99 (0.89, 1.11)	0.11
70	Neyagawa	0.98 (0.93, 1.03)	0.50
71	Takatsuki	1.07 (1.00, 1.13)	0.38
72	Kobe	1.03 (1.00, 1.05)	2.17
73	Himeji	1.03 (0.99, 1.07)	0.89
74	Akashi	1.04 (1.00, 1.09)	0.76
75	Kakogawa	1.02 (0.98, 1.06)	0.81
76	Amagasaki	1.05 (0.99, 1.12)	0.34
77	Takarazuka	1.06 (0.94, 1.19)	0.10
78	Nara	1.02 (0.98, 1.06)	0.82
79	Wakayama	1.01 (0.98, 1.05)	1.05
80	Okayama	0.99 (0.97, 1.01)	1.98
81	Kurashiki	1.01 (0.98, 1.04)	1.37
82	Hiroshima	1.01 (0.99, 1.03)	2.11
83	Fukuyama	1.04 (1.01, 1.07)	1.48
84	Kure	0.95 (0.88, 1.02)	0.28
85	Shimonoseki	1.00 (0.96, 1.03)	1.16
86	Tokushima	0.98 (0.94, 1.03)	0.67
87	Takamatsu	1.04 (1.01, 1.07)	1.44
88	Matsuyama	1.02 (0.99, 1.06)	1.22
89	Kochi	1.01 (0.97, 1.05)	0.70
90	Fukuoka	0.98 (0.96, 1.00)	2.92
91	Kitakyushu	1.00 (0.98, 1.01)	3.22
92	Kurume	0.99 (0.95, 1.03)	0.79
93	Saga	1.01 (0.96, 1.05)	0.80
94	Nagasaki	0.98 (0.93, 1.03)	0.53
95	Sasebo	1.00 (0.97, 1.04)	1.00
96	Kumamoto	0.99 (0.96, 1.02)	1.28
97	Oita	1.00 (0.97, 1.04)	1.15
98	Miyazaki	0.99 (0.94, 1.04)	0.55
99	Kagoshima	1.04 (1.01, 1.07)	1.35
100	Naha	0.94 (0.84, 1.05)	0.12
Overall (I-squared = 9.8%, p = 0.215)		1.01 (1.01, 1.02)	100.00

NOTE: Weights are from random effects analysis

eFigure 3. City-specific odds ratios (ORs) of total non-accidental death with a 10 μg/m^3 increase in coarse particulate matter concentrations at lag0-1

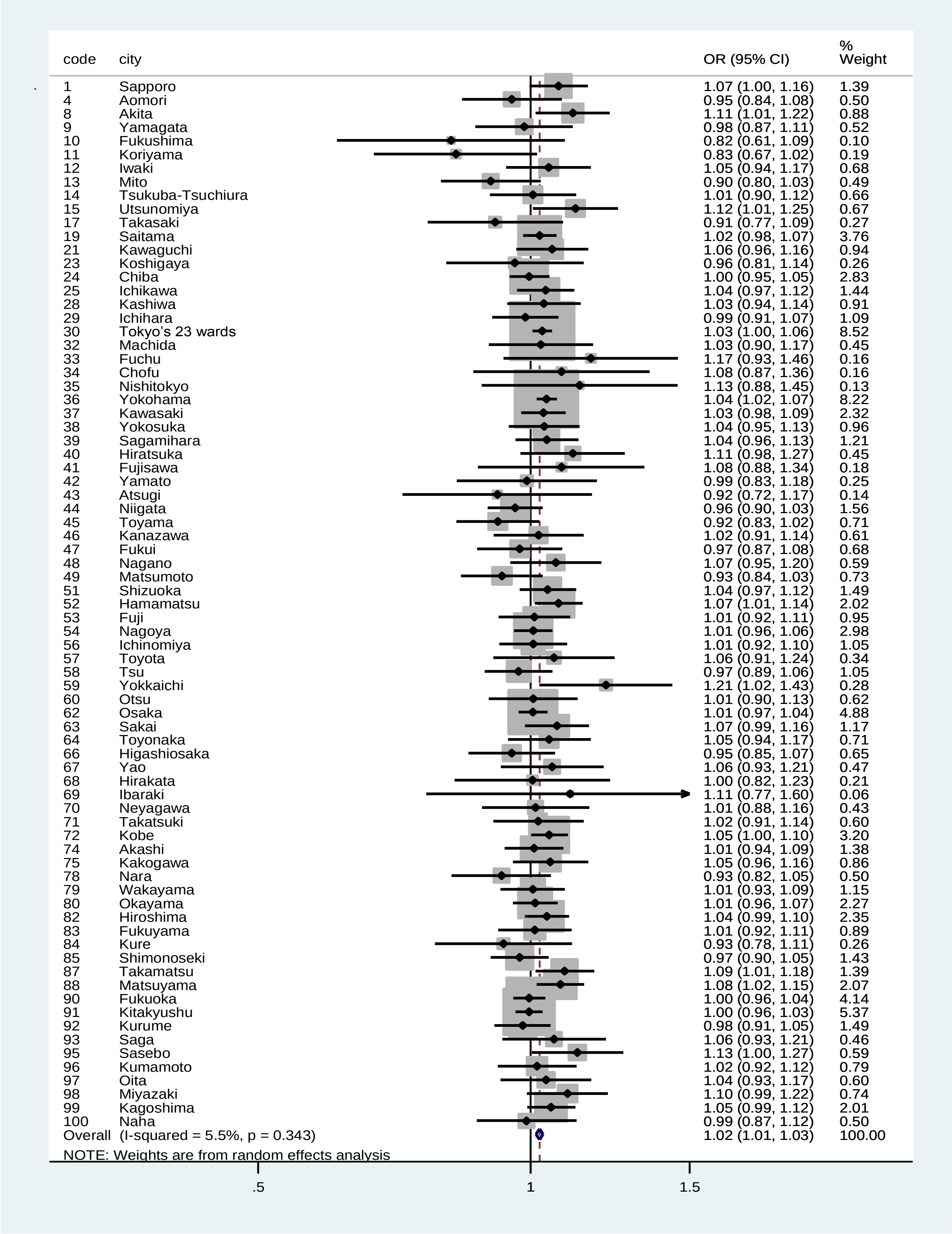

eFigure 4. Sensitivity and stratified analyses of the association between coarse PM exposure and total non-accidental mortality

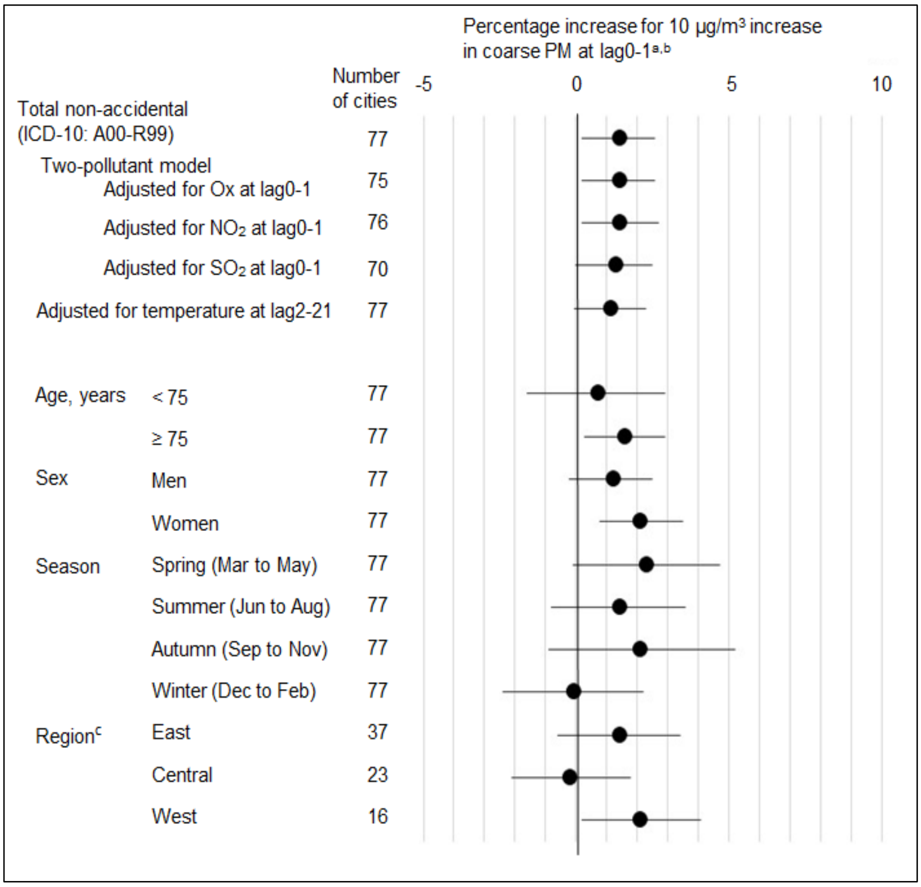

CI, confidence interval; ICD, international classification of diseases; NO_2, nitrogen dioxide; Ox, photochemical oxidants; PM, particulate matter; SO_2, sulphur dioxide.

[a]Concentrations of coarse PM were calculated by subtracting $PM_{2.5}$ concentrations from those of suspended particulate matter.

[b]Adjusted for $PM_{2.5}$ at lag0-1, coarse PM at lag2, and lag3, ambient temperature at lag0-1, relative humidity at lag0-1, and influenza epidemics.

[c]See eTable 1.
